# The Kesty Redness Scale: A Pilot Validation Study for a Novel Tool for Evaluating Facial Redness in Cosmetic and Clinical Dermatology

**DOI:** 10.1111/jocd.70039

**Published:** 2025-04-01

**Authors:** Chelsea E. Kesty, Katarina R. Kesty

**Affiliations:** ^1^ St. Petersburg Skin and Laser St. Petersburg Florida USA; ^2^ Kesty AI St. Petersburg Florida USA

**Keywords:** facial redness, laser resurfacing, rosacea, ruby laser, skin resurfacing, vascular

## Abstract

**Background:**

Facial redness is a common concern in dermatology, affecting patients with conditions such as rosacea, post‐inflammatory erythema, and other vascular irregularities. Despite its prevalence, existing tools for quantifying facial redness are limited in their clinical utility and ease of use.

**Aims:**

To develop a high‐performing redness scale.

**Methods:**

This study introduces the Kesty Redness Scale (KRS), outlines its development and validation process, and discusses its potential clinical applications.

**Results:**

The investigators rated the scale as useful and easy to use, and the majority stated they would use it in clinical practice to document patient characteristics. The results of the evaluation utilizing Gwet's AC2, Kendall's W, Spearman's ρ, Weighted Cohen's kappa, and Bland–Altman analysis —showcasing strong ordinal agreement, robust rank concordance, and negligible bias—demonstrate that this new rating system is both reliable and valid for measuring skin hyperpigmentation on a 0–3 scale.

**Conclusions:**

The KRS is a reliable, easy‐to‐use tool that enhances the assessment of facial redness in dermatology. Its validation through expert evaluation and statistical analysis underscores its potential to improve clinical practice and research.

## 1 Introduction

Facial redness is a common concern in dermatology, affecting patients with conditions such as rosacea, post‐inflammatory erythema, and other vascular irregularities [1, 2]. In cosmetic dermatology, facial redness due to sun exposure, acne, rosacea, and other skin conditions is a common presenting complaint during a cosmetic consult [3, 4, 5]. Lasers and combinations of lasers treat redness, and a scale to quantify the before and after change in redness would be useful for cosmetic dermatology and lasers. Despite its prevalence, existing tools for quantifying facial redness are limited in their clinical utility and ease of use. Most scales for redness are tailored to disease states such as rosacea, and thus are limited in the cosmetic dermatology and laser application [6, 7, 8]. To address this gap, we developed the Kesty Redness Scale (KRS), a five‐point ordinal scale for assessing facial redness severity (Table 1). This study aimed to validate the KRS using expert evaluations of clinical images and to explore its clinical utility.

**TABLE 1:** Kesty redness scale (KRS).

Grade	Description	Examples
0	Clear	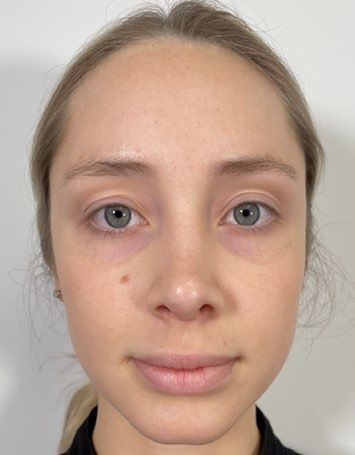
1	Almost clear. Some mild but almost imperceptible redness covering less than 10% of facial surface area	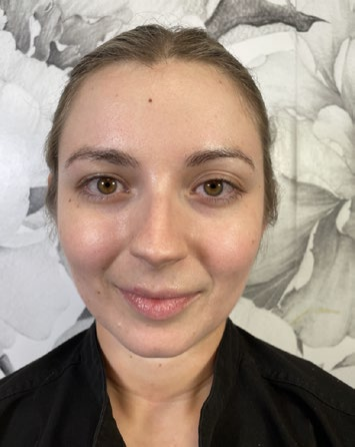
2	Mild, somewhat noticeable redness covering 10%–25% of facial surface area	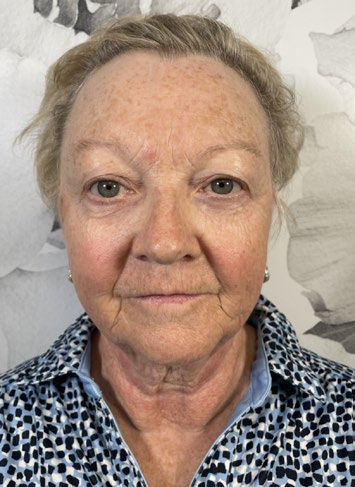
3	Moderate, noticeable redness covering 25%–50% of facial surface area	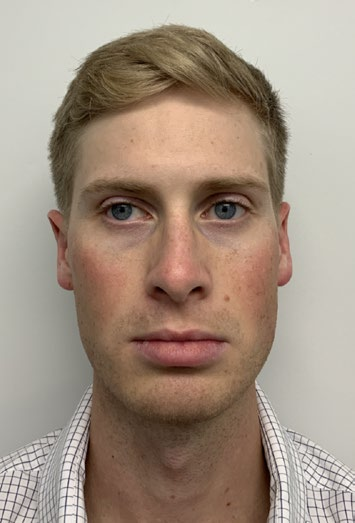
4	Severe redness that distracts from facial features and covers > 50% of facial surface area	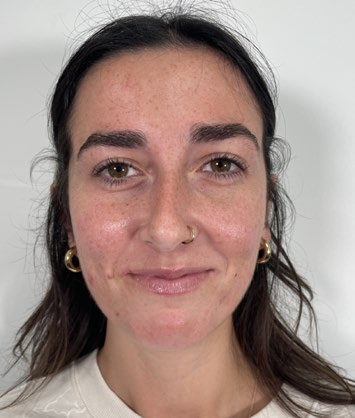

## 2 Methods

This prospective observational study was conducted to evaluate the inter‐rater reliability and clinical applicability of the KRS. Ten esthetic professionals, including board‐certified dermatologists, board‐certified plastic surgeons, and others in esthetics, participated as evaluators. More than 100 photographs of faces of volunteer subjects were compiled. All were taken with the patient facing forward or turned but both eyes were visible. The median age of the participants was 48 (range 31–65). The images represented a range of redness severities associated with different dermatological conditions and cosmetic treatment outcomes. A study team, comprised of a physician, selected five photographs as representing the five severity scales on the KRS. A written description of each level on the scale was written (Table 1). Photographs were chosen based on the ability of each photograph to represent the level on the scale as well as an equal difference between levels (e.g., the difference between level 1 and 2 is the same as between level 2 and 3). This set of reference photographs and written descriptions was used to rate the photographs in the set of photographs. The reference photographs as well as the written scale was sent to the participants in the study. A set of 20 unlabeled photographs was also sent and participants were asked to rank the photographs according to the KRS. The participants were also asked “Is the scale easy to use?” (Yes/No), and “Would you use this scale as part of your clinical practice?” (Yes/No).

## 3 Statistical Methods

This study focused on evaluating a new method for rating facial skin redness (Rater: Kesty) and comparing its performance to that of a group of expert raters (Raters: B–L) using a 0–4 ordinal scale. A variety of statistical tools were employed to assess both overall agreement across all raters and pairwise alignment between the novel approach and the experts. The findings demonstrated that the novel method performs at a high level of accuracy and consistency, aligning closely with industry expectations.

## 4 Overall Measures of Agreement

### 4.1 Gwet's AC2

To measure inter‐rater consistency, we applied Gwet's AC2, a statistical measure specifically designed for ordinal data that accounts for chance agreement. The categories set was $\left\{1,2,\ldots ,R\right\}$ and defined quadratic weights were:

$$W\left(c_{i},c_{j}\right)=1-{\left(\frac{\left|c_{i}-c_{j}\right|}{R-1}\right)}^{2}$$

The observed agreement Po for N items, each with K raters, is computed by considering category frequencies fc per item and forming pairwise proportions pci,cj. Expected agreement Pe is derived from the marginal category probabilities pc. Gwet's AC2 is given by:

$$\mathrm{AC}2=\frac{P_{o}-P_{e}}{1-P_{e}}$$

Gwet's AC2 was calculated to be approximately 0.9207, highlighting an excellent degree of agreement among raters.

### 4.2 Kendall's W

Rank agreement was further explored using Kendall's W, which assesses how consistently raters ranked items. A value nearing 1 indicated that the raters displayed strong alignment in their rankings, reflecting excellent consistency in evaluating skin pigmentation.

For *n* items and *m* raters, let Rij be the rank of the i−th item by the j−th rater.

Define $R_{i}=\sum_{j=1}^{m}R_{\mathrm{ij}}\text{and}\bar{R}=\frac{1}{n}\sum_{i=1}^{n}R_{i}$.

Kendall's W is given by:

$$W=\frac{12\sum_{i=1}^{n}{\left(R_{i}-\bar{R}\right)}^{2}}{m^{2}\left(n^{3}-n\right)}$$

A value of W=0.8839 suggests a high degree of consistency in the ranking of items across raters.

## 5 Pairwise Measures of Association and Agreement

### 5.1 Spearman's Rank Correlation

The monotonic relationships between the novel method and individual expert raters were analyzed using Spearman's rank correlation (*ρ*). For *n* items, let di=Ri1−Ri2 be the difference in the ranks assigned by the two raters. Spearman's *ρ* is given by:

$$\rho =1-\frac{6\sum_{i=1}^{n}d_{i}^{2}}{n\left(n^{2}-1\right)}$$

Values at 0.90 or above consistently indicate an extremely strong monotonic relationship (Table 2).

**TABLE 2:** Spearman's rank correlation (*ρ*).

Rater pair	Spearman's *p*
*Kesty—B*	0.9498
*Kesty—C*	0.9472
*Kesty—D*	0.9522
*Kesty—E*	0.8975
*Kesty—F*	0.9481
*Kesty—G*	0.8978
*Kesty—H*	0.8522
*Kesty—I*	0.9074
*Kesty—J*	0.9235
*Kesty—K*	0.9330
*Kesty—L*	0.9353

Rater	Bias	Lower limit	Upper limit
*B*	−0.1579	−1.1408	0.8250
*C*	0.2105	−0.8387	1.2597
*D*	0.1053	−1.0063	1.2168
*E*	0.3158	−0.9994	1.6310
*F*	−0.3158	−1.2518	0.6202
*G*	0.1053	−1.1841	1.3946
*H*	0.2632	−1.5664	2.0927
*I*	0.1053	−1.3402	1.5507
*J*	−0.0526	−1.2703	1.1650
*K*	0.0000	−1.1316	1.1316
*L*	0.0526	−1.1650	1.2703

### 5.2 Weighted Cohen's Kappa

For direct comparisons on categorical agreement, we used Weighted Cohen's kappa. Let Oij be the observed proportion of assignments where Rater A and another rater choose categories i and j, and let Eij be the expected proportion under independence. Using the same quadratic weights $\mathrm{wij}=W\left(c_{i},c_{j}\right)$ defined above, weighted kappa is:

$$\kappa_{w}=\frac{\sum_{i,j}w_{\mathrm{ij}}O_{\mathrm{ij}}-\sum_{i,j}w_{\mathrm{ij}}E_{\mathrm{ij}}}{1-\sum_{i,j}w_{\mathrm{ij}}E_{\mathrm{ij}}}$$

The results of our study included weighted kappas frequently above 0.90, indicating that the novel method's categorical assignments closely align with those of the experts (Table 3).

**TABLE 3:** Weighted Cohen's kappa.

Rater pair	Weighted Cohen's kappa
*Kesty—B*	0.9474
*Kesty—C*	0.9434
*Kesty—D*	0.9412
*Kesty—E*	0.9000
*Kesty—F*	0.9412
*Kesty—G*	0.9184
*Kesty—H*	0.8046
*Kesty—I*	0.9020
*Kesty—J*	0.9293
*Kesty—K*	0.9388
*Kesty—L*	0.9278

### 5.3 Bias and Limits of Agreement

Finally, a Bland–Altman analysis was conducted to explore potential systematic bias. For two raters, define the difference Di=Ri1−Ri2 and the average $\mathrm{Ai}=\left(\mathrm{Ri}1+\mathrm{Ri}2\right)/2$. The mean difference (bias) and the standard deviation (SD) of differences provide limits of agreement (LoA):

$$\text{Bias}=\frac{1}{n}\sum_{i=1}^{n}D_{i},\mathrm{LoA}=\text{Bias}\pm 1.96\cdot \mathrm{SD}$$

Minimal bias and narrow LoA were apparent after our analysis, suggesting no substantial systematic deviation of the novel method's scores from those of industry experts. Although Bland–Altman is more commonly applied to continuous data, it still provides a useful check for consistent over‐ or underestimation, which was not evident here.

## 6 Results

To summarize, the evaluation utilizing Gwet's AC2, Kendall's W, Spearman's *ρ*, Weighted Cohen's kappa, and Bland–Altman analysis offers a well‐rounded assessment of the novel method's performance. The results—showcasing strong ordinal agreement, robust rank concordance, and negligible bias—demonstrate that this new rating system is both reliable and valid for measuring skin hyperpigmentation on a 0–3 scale. Furthermore, these findings establish the method as a credible and industry‐aligned tool. Notably, 100% of participants found the scale easy to use, with all users expressing their willingness to incorporate it into their clinical workflows.

## 7 Discussion

The number of cosmetic procedures in the United States increases every year. One of the fastest‐growing procedures within cosmetics is lasers for rejuvenation. Although patient satisfaction is important in lasers and cosmetics, a clinically useful scale to objectively evaluate the results of laser treatments can help the doctor–patient interaction. Having various scales for redness can help the evaluator choose the appropriate one for the patient, as some are more appropriate for medical conditions and others may be tailored to cosmetic concerns, such as the KRS [6, 9, 10, 11, 12, 13]. The KRS provides a standardized framework for evaluating facial redness in cosmetic dermatology, addressing a significant unmet need in both cosmetic and clinical dermatology. Its simplicity and reliability make it an ideal tool for use in diverse clinical settings. Potential applications include in cosmetic procedures. The KRS can objectively quantify redness before and after treatments such as laser therapy, enabling clinicians to document treatment outcomes and improve patient satisfaction.

Statistical validation of scales is the current gold standard for approval and acceptance of clinical scales. Despite this, the reliance on a human's visual assessment can introduce subjectivity that may be influenced by the evaluator's training, experience, and personal biases. An ideal situation would be a non‐biased and non‐human large language model based on artificial intelligence that “learns” the scale and is rigorously tested; then it itself rates patient photographs. An artificial intelligence model can help eliminate human subjectivity and would be extremely helpful in both clinical and research applications. Further studies can include a larger sample size of evaluators as well as more patient images for classification, which can further support the use of the KRS across a wide demographic of the population. Studies with a larger sample size of evaluators and patient images can be done to improve the statistics that support the scale's reliability and ease of use. Additional patient photographs, including patients that have facial erythema from a wide variety of dermatologic conditions, including acne, rosacea, post‐inflammatory, lupus, sun damage, and others, can also be included in further trials on the KRS to support the scale's applicability to all causes of facial redness.

Further use of the KRS can include implementing the scale in clinical trials. The scale can serve as an endpoint for studies investigating lasers and other treatments for conditions like poikiloderma, post‐acne erythema, and photodamage. This scale can also facilitate improved doctor–patient communication by providing an intuitive structure that facilitates discussions with patients about the severity of their condition and the expected outcomes of treatment.

## 8 Conclusion

The KRS is a reliable, easy‐to‐use tool that enhances the assessment of facial redness in dermatology. Its validation through expert evaluation and statistical analysis underscores its potential to improve clinical practice and research. Future studies may focus on the application of scales in artificial intelligence models to minimize dependence on human evaluators.

## Author Contributions

K.R.K. and C.E.K. conceived the study, wrote and revised the manuscript, and funded the study. All authors have reviewed and approved the article for submission.

## Conflicts of Interest

The authors declare no conflicts of interest.

## Data Availability

The data that support the findings of this study are available on request from the corresponding author. The data are not publicly available due to privacy or ethical restrictions.
